# What will it take? Pathways, time and funding: Australian medical students’ perspective on clinician-scientist training

**DOI:** 10.1186/s12909-017-1081-2

**Published:** 2017-12-08

**Authors:** Diann S. Eley, Charmaine Jensen, Ranjeny Thomas, Helen Benham

**Affiliations:** 1 Faculty of Medicine, The University of Queensland Office of Medical Education, 288 Herston Road, Brisbane, 4006 Queensland Australia; 2 The University of Queensland, Translational Research Institute, Australian Academy of Health and Medical Sciences, Princess Alexandra Hospital, Woolloongabba, Australia; 3 Department of Rheumatology, The University of Queensland, Diamantina Institute, Princess Alexandra Hospital and Translational Research Institute, Woolloongabba, Australia

**Keywords:** Medical students, MD-PhD, Clinician-scientist, Research training, Training pathways, Research career decision making, Barriers to research careers

## Abstract

**Background:**

Clinician-scientists are in decline worldwide. They represent a unique niche in medicine by bridging the gap between scientific discovery and patient care. A national, integrated approach to training clinician-scientists, typically programs that comprise a comprehensive MD-PhD pathway, are customary. Such a pathway is lacking in Australia. The objective was to gather perceptions from Australian medical students on factors they perceive would influence their decision to pursue clinician-scientist training.

**Methods:**

A cross-sectional mixed methods design used quantitative and qualitative questions in an online self-report survey with medical students from a four-year MD program. Quantitative measures comprised scaled response questions regarding prior experience and current involvement in research, and short- and long-term opinions about factors that influence their decisions to undertake a research higher degree (RHD) during medical school. Qualitative questions gathered broader perceptions of what a career pathway as a clinician-scientist would include and what factors are most conducive to a medical student’s commitment to MD-PhD training.

**Results:**

Respondents (*N* = 418; 51% female) indicated Time, Funding and Pathway as the major themes arising from the qualitative data, highlighting negative perceptions rather than possible benefits to RHD training. The lack of an evident Pathway was inter-related to Time and Funding. Themes were supported by the quantitative data. Sixty percent of students have previous research experience of varying forms, and 90% report a current interest, mainly to improve their career prospects.

**Conclusions:**

The data emphasise the need for an MD-PhD pathway in Australia. A model that provides an early, integrated, and exclusive approach to research training pathways across all stages of medical education is suggested as the best way to rejuvenate the clinician-scientist. A national pathway that addresses factors influencing career decision making throughout the medical education continuum should include an appropriate funding structure, and provide early and continuing advice and mentoring. It should be flexible, gender equitable, and include post-graduate training. The implications of implementing MD-PhD programs represent a substantial investment. However this should not be a deterrent to Australia’s commitment to an MD-PhD pathway, but rather a challenge to help ensure our future healthcare is guided by highly trained and competent clinician-scientists.

## Background

The decline in the number of clinician-scientists is documented worldwide with the earliest forewarnings in the late 1970s [1]. While many clinicians undertake research, a clinician-scientist is a clinically trained health professional with additional training in research, typically a PhD. Strategies to reverse this trend acknowledge that nurturing new generations of clinician-scientists should start early, and certainly during medical school [2–4].

The earliest MD-PhD program started in the United States (US) at Johns Hopkins University in the 1950s, and led to the prestigious National Institutes of Health (NIH) Medical Scientist Training Program (MSTP) [5] in 1964. Similar programs exist in several countries that focus on both undergraduate and/or postgraduate training. Recently, financial pressures have impacted even the well-established MSTP [6, 7], and most recently, Canada experienced cut-backs to their Canadian Institutes of Health Research clinician-scientist training program [8]. Nevertheless, these programs continue to provide a tangible and prestigious pathway for research-focussed students, to be trained at the finest research institutions by leading clinician-scientists. Such schemes are lacking in Australia.

There is much literature devoted to how educators might inspire and engage medical students to increase the numbers who choose to follow a clinician-scientist career. There has been attention given to what students understand about research and how to incorporate it into their clinical career. Students who gain research experience during medical school were found to publish more frequently after graduation [3]. For females, research experience [9], and quality mentorship [10] as part of the curriculum is an even more important predictor of future research activity. This is significant, given the recognised gender issues that impact on females’ commitment to the extra training time required to complete an MD-PhD [11].

Krupat [9] demonstrated the negative predictors of intensive research involvement included being female, financial/debt concerns, and minority status. Mentors were a strong predictor for future research involvement, especially for females. A recent multi-institutional study across five countries, which did not include Australia, found only 43% of students agreed that they receive sufficient research training during medical school [12]. The barriers to research involvement were time; (during medical school, and prolonged time for a research degree); lack of funding and increasing debt; and finding mentors.

In Australia there is no structured framework for clinician-scientist training although most schools offer an MPhil or PhD intercalated with the medical program. There are few data on the numbers and progression of medical students who undertake a concomitant MPhil or PhD. Nevertheless, there is high interest in research among Australian medical students, which increases through to early post-graduate years [13, 14].

Whilst several studies have quantified medical student perceptions about research [12, 15–18], the current study aimed to add an Australian perspective through student opinions on how Australia might consider a national approach to its decline in clinician-scientists. The research questions addressed: 1) what factors influence student interest in combining research with their medical program, 2) are there facilitators and barriers to pursuing a research career alongside medical training, and 3) are there solutions and recommendations Australia might consider to support the training of more clinician-scientists?

## Methods

Ethics was approved by The University of Queensland Human Research Ethics Committee approval number 2013000495. Participants provided written consent, which was documented on the questionnaire and approved by this ethics committee.

### Design

A cross-sectional mixed-methods embedded design comprised an online questionnaire incorporating qualitative items (free-response written comments) with quantitative items (Likert scale questions), in order to provide context to quantitative response patterns. The questionnaire was piloted previously with recent post-graduate students for comprehension.

### Participants and setting

Data was collected in 2015 at The University of Queensland’s Faculty of Medicine. The University is considered a research intense institution and has a total enrolment of over 51,000 students and the Medical Program has an average yearly intake of approximately 500 students in the MD degree. All medical students across the four-year medical program were sent an invitation via the School’s student community website containing an online link (Survey Monkey©) to the questionnaire. One reminder was sent. The medical student community website is used for extra-curricular announcements and invitations to surveys in order to reduce the number of emails that students receive. Approximately two thirds of the student cohort access this site regularly and engage with the content.

### Analysis

Quantitative data was analysed using SPSS 24 to describe the sample demographics, (age, sex, domestic/international status, year of study, prior research experience), and to quantify qualitative responses to opinion items regarding facilitators/barriers to combining research with medical school and/or early post-graduate training. Qualitative data was analysed using NVivo qualitative data analysis Software (QSR International Pty Ltd. Version 10, 2012). An inductive approach to thematic analysis [19] was used to code student written responses. The initial analysis was performed by CJ and identified broad themes within all the data, followed by a more focused analysis carried out by HB and DE to identify sub-themes. Final thematic structure was determined when investigators’ reached consensus on coding decisions. Representative quotes are presented with no identifying demographics.

## Results

An accurate response rate is not possible because as described above, the MD Program’s community website reliably reaches two-thirds of all students. Nevertheless, a total of 418 students responded, giving a response rate of 32%. The student sample population is representative of all other Australian medical schools. Most were first year students (*n* = 173; 43%), with decreasing participation in second *(n* = 89; 22%), third (*n* = 76; 19%), and fourth years (*n* = 62; 16%). The majority (*n* = 245; 61%) were domestic students, female (*n* = 204; 51%), and aged between 21 and 54 years (mean = 26.54).

### Question 1: Factors that influence interest in combining research with medical school

Students’ research experience prior to medical school included Honours (20%; *n* = 74), research higher degrees (RHD) (MPhil 12%, *n* = 46; PhD 3%, *n* = 11), and casual/voluntary experience which made up 35% (*n* = 133). Forty percent (*n* = 155) of students reported no previous involvement in research. The majority (70%, *n* = 272) were not currently involved in research alongside the medical program, although 20% (*n* = 80) reported doing casual/voluntary research and 9% (*n* = 12) were formally enrolled in RHDs. Ten percent (*n* = 41) of students indicated little/no interest in gaining research experience.

The remaining students were either thinking about (36%, *n* = 144), or definitely planning on (32%, *n* = 128) getting involved in research at some point in their medical degree. Table 1 lists reasons for this involvement. A small percentage has a strong interest in a particular research area, and believe research will help to become a better clinician. The biggest reason was to improve their CV and the chances of entering a preferred speciality. This was acknowledged in several student comments, for example:

“*Not all research is done with patients in mind. Some are done purely to improve their chances of getting into a training program.” [male-year 1].*

**Table 1:** Main reasons for students’ interest in combining research with their medical program

Multiple-choice response to the question^a^: *What is the main reason for your interest in combining research with your medical degree?*	Number	Percent
It will improve my CV and chances of getting into my preferred registration discipline/college	172	41.15
I have a strong interest in a particular research area	51	12.20
I believe it will help me be a better clinician	51	12.20
I want to develop some research skills	41	9.81
I am interested in an academic career in the future	34	8.13
I hope to get some research output such as co-authorship on a paper or conference abstract	23	5.50
Total	372	89

^a^Only one choice was permitted

### Question 2: Facilitators and barriers to pursuing a research career

Table 2 lists the factors in the short-term, (during medical school and through postgraduate years 1-4), and long-term (registrar training and beyond), that would influence students’ pursuit of research training during medical training. In the short–term, students felt that job prospects, professional advancement and career options would be improved. While income would not be affected, they felt life-balance would be much worse, and career autonomy unchanged. In the long-term, students thought income, life-balance and career autonomy would improve somewhat. Job prospects, career options and professional advancement would continue to be much improved.

**Table 2:** Rating^a^ of factors in the short-term (during medical school and through postgraduate years 1-4), and long-term (registrar training and beyond), that would improve or worsen by pursing research during medical school

*Please choose to what degree you feel the following factors would be improved or worsened by pursuing research during your medical training.*	Much worse % (*n*)	Worse % (*n*)	Neither worse nor improved % (*n*)	Improved % (*n*)	Much improved % (*n*)
Job prospects					
Short-term	0.3 (1)	1.0 (4)	20.0 (76)	60.0 (214)	21.0 (81)
Long-term	0.0 (0)	0.5 (2)	10 (37)	55 (207)	35 (131)
Income					
Short-term	1.0 (5)	7.0 (25)	72.0 (269)	17.0 (64)	3.5 (13)
Long-term	0.0 (0)	3.99 (15)	36.97 (139)	181 48.14%	41 10.90%
Professional advancement/opportunities					
Short-term	0.0 (0)	0.5 (2)	12.5 (47)	62.7 (236)	24.2 (91)
Long-term	0.0 (0)	0.5 (2)	8.5 (32)	60.1 (226)	30.9 (116)
Personal life balance					
Short-term	13 (49)	56.8 (214)	23.9 (90)	5.6 (21)	0.8 (3)
Long-term	9.0 (34)	32.4 (122)	44.0 (166)	11.4 (43)	3.2 (12)
Career independence/autonomy					
Short-term	0.8 (3)	5.8 (22)	48.8 (184)	39.8 (150)	4.7 (18)
Long-term	0.0 (0)	3.7 (14)	34.8 (131)	49.3 (186)	12.2 (46)
Career options					
Short-term	0.3 (1)	1.3 (5)	17.2 (65)	64.2 (64)	17.0 (64)
Long-term	0.3 (1)	0.8 (3)	10.6 (40)	63.0 (237)	25.3 (95)

^a^Ratings were on a 5 point Likert scale from 1 = much worse to 5 = much improved

**Fig. 1:**
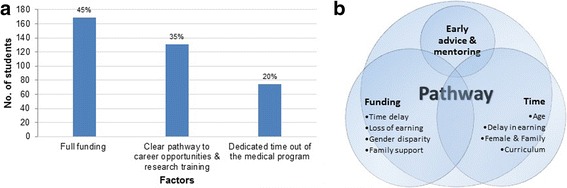
**a** shows the response to the question, “What one major factor would encourage you to get involved in research during medical school?” **b** depicts the interrelation of the major themes identified in the qualitative data

When asked “What one major factor would encourage you to get involved in research during medical school?” funding was the biggest facilitator followed by a clear pathway and dedicated time for research. See Fig. 1a.

While a very small number of students definitively did not want to pursue any form of research, the majority of qualitative responses were mixed and often prefaced with “*I would do research if*….” thereby qualifying moderating factors such as barriers and facilitators. Generally, students did not perceive becoming a clinician-scientist as a straightforward path.

Table 3 presents the major themes identified from the qualitative data as ‘Time’, ‘Funding’, and ‘Pathway’ which were largely portrayed as obstacles to undertake research during medical school. Importantly, there was considerable interaction between the themes. See Fig. 1b.

**Table 3:** Thematic summary of qualitative responses to open-ended questions about pursuing a clinician-scientist career (*N* = 263; total number of individual open-ended responses)

Major themes and sub-themes	Representative quotes	^*a*^ *n*, %
TIME as a major theme was mainly about the extra time doing research might add on to medical training but had ramifications around delayed earnings, starting a family and the best use of time in the medical curriculum.		79, 30%
Time relating to Age	*“The thought of taking 2 years out while a student to defer income another 2 years while currently struggling financially is off putting”* [M-2] *“Being a mature-age student, adding extra time onto an already lengthy training process without substantial financial recompense is not worth the time investment.”* [M-2] *‘“Depending on your age, I believe the answer to this question changes. The older you are, the less inclined you are to extend the length of your studies, particularly due to cost.”* [F-3] *“The additional years needed are the only reason I am not seeking to complete a RHD.”* [M-2]	
Time relating to females and starting a family	*“As a female student who wants to potentially have a family as well as a full-time career one day, I already feel the pressures of a long training program ahead.”* [F-1] *“I am prioritising in being as advanced through my degree as possible before I am required to take time out to have a family. Adding two or more years to my medical school seems like it makes this more difficult.”* [F-2] *“Mostly because of my age. I am 33 this year and I want to start a family. I am uncertain how I would be able to manage this with other professional commitments.”* [F-1]	
Time relating to the medical curriculum and limiting opportunities	*“The time commitments for med school is the biggest limiting factor for me pursuing research during this time.”* [M-1] *“Research is such a great compliment to our medical degrees. However, time pressures seem to be a major deterrent for those interested in it.”* [F-2] *“To try fit in research while already not having enough time to practice and apply what is being taught in Clinical Science and Examination is a huge negative deterrent from doing any research while studying.”* [F-2]	
FUNDING was closely related to the extra time required and potential loss of earning, and the impact on family plans.		63, 24%
Extra time and delay or loss of earning a salary	*“As a 21-year-old student who supports themselves financially, it is difficult for me to find the time for Medical school, a part time job and research. Financial incentives would definitely influence my opinion.”* [M-4] *“Another two years working on PhD means two years of lost income over my lifetime, AS WELL AS two years of more interest accruing on my already massive debt.”* [M-1] *“I can’t see research ever being a viable option other than as a passion simply because of the length of time undergraduate and medical school takes and the lack of income options.”* [F-3] *“The only real barrier to me pursuing it in my medical degree is that I cannot financially afford to extend my study period.”* [F-1]	
Funding to encourage and support students with families, especially females	*“I am 31, I have 4 children and so I feel the need to get into paid work ASAP.”* [M-3] *“As a 1st year medical student who will be turning 30 this year, it is definitely an influence. I have to start thinking about family planning immediately after graduation (main concern).”* [F-1]	
PATHWAY as a major theme comprises all of the above themes and comments as well as recommendations to provide early advice and mentorship to students.		92, 35%
A clear pathway to career opportunities beyond medical school	*“Getting funding is a major factor with regards to committing to clinician scientist pathway.”* [M-1] *“The one thing that would be encouraging is a defined path(s) for a clinician-scientist route when starting internship.”* [M-3] *“Some form of guarantee that time and funding would be made available goes a long way towards alleviating some of the fundamental burdens of students.”* [F-1] *“It would be nice to be able to see more of this in action and have a structured pathway to follow.”* [F-4] *“A more organized pathway connecting students to research and at least some funding to help supplement medical training.”* [M-4] “*Not every clinician will be a researcher (and* vice versa*). Those that have the talent and “fire breathing within” for a clinician-scientist pathway should be freely allowed to follow that path. And should be helped/mentored to do so along the way.”* [M-1]	
Mentoring and early career advice	*“A major obstacle for most students is taking on a research load on top of medical school. Information on support and process of this would help encourage students.”* [F-2] *“A close mentor who could guide me through the research process would be beneficial.”* [F-1] *“I think a mentoring program, in which students can meet and discuss options with students that are currently or have in the past done research during their time at UQ - would be of great help.”* [M-2]	

^*a*^ n = number of responses relating to that theme, % = those responses as a percentage of the total 263 open-ended responses

Demographics are contained in brackets after quote as [sex-year of study] where male = M; female = F, and year of study = 1,2,3,4]

‘Time’ was identified as one of the biggest barriers to undertaking a RHD during medical school. The additional training time was reported by 81% (*n* = 306) of respondents as a negative/strongly negative influence. ‘Time’ was inter-related to several factors. One was ‘Age’ as reflected in this comment:

*“The additional years needed are the only reason I am not seeking to complete a RHD.” [female-year 2]*

*“The impact of the extra two years is, for me, a consideration not just for myself, but for my partner, who has had to make choices regarding his career as a result.” [female-year 3]*

‘Age’ was in turn more specifically related to comments associated with ‘Family’ and was most often, but not exclusively, noted by female’ students. Female (50%) responses showed research interest in similar proportions to males. However, many comments related to barriers specific to females, suggesting that fewer females will pursue research involvement in medical school leading to a career as a clinician-scientist.

*“As a 25-year-old woman who wants a family, I'm very aware that adding years to my junior training is not conducive to obtaining a fellowship before my fertility becomes a gamble. Time is a valued commodity.” [female-year 3]*

*“The impact that research would potentially have on delaying completion of training and thus reducing the already limited years available for having children seems small but is a big deal to me. I have talked to several male students about this and it has never been a consideration or issue for any of them”. [female-year 1]*

Time was also related to the medical ‘Curriculum’:

*“The strongest limiting factor to me undertaking research during my MD is the lack of sufficient time relative to coursework load. However, I do not want to postpone/pause the degree in order to accommodate this.” [male-year 1]*

Students acknowledged the association and dilemma of financial support and the constraints of the curriculum.

*“It's a close call between some form of financial support or dedicated time away from the MD program. I can't imagine being able to fit substantial research time on top of the current workload.” [male-year 1]*

‘Funding’ was identified as a major theme in its own right, but it was difficult to separate from time and family. One student summarised this well:

*“Funds are definitely helpful, however, without the appropriate amount of time to actually do the research, it would be moot.” [male-year 2]*

‘Funding’ was reported by 45% (*n* = 170) of our sample as the single most important factor to encourage research activity during medical school. The clearest connection was the extra time and consequent delay in earning a salary.

*“Some students I've met are interested in research but are also starting/supporting a family, hence they would need a source of income in order to support their families.” [female-year 1]*

*“Funding is the main issue for me. With the debts from my undergrad and the climbing debts of my MD, it is already hard to commit to further debt for an MPhil, even more so for taking 2 years off for a PhD”. [female-year 2]*

### Question 3: Solutions Australia might consider to support training of more clinician-scientists

‘Pathway’ as a major theme, was inter-related to both ‘Time’ and ‘Funding’ and encompassed the dominant issues that were expressed by the students. Sixty-five percent (*n* = 306) of respondents reported that having a clear and practical career pathway, offering protected research time and funding would alter their decision (positively) to commit to research training.

*“I think making pathways clearer, and having people work towards them from the start of their medical degree would be very helpful.” [female-year 4]*

More students preferred a pathway, if available, during medical school (28%; *n* = 105), versus at graduation (18%; *n* = 67), or from completion of intern training (21%; *n* = 78). For example:

*“I thoroughly enjoy research and would love to pursue a career that mixes research and clinical work. I would find it extremely appealing if there was a defined track from finishing medical school.” [male-year 2]*

Survey data and qualitative comments highlighted the need for commitment and consideration by medical schools to provide early advice and mentorship to those who genuinely feel a desire to follow a clinical-research career.

*“It is so difficult to determine, at such an early stage in year 1 of MD, if I'm prepared to add more time onto my studies, without knowing what direction my career will go.” [female-year 1]*

## Discussion

This study used a mixed-methods design to gather perceptions from a sample of Australian medical students regarding research training during medical training. It addresses the lack of an Australian perspective in the current literature, with which it is consistent in many ways.

One similarity, shared worldwide, is that most medical students are interested in and pursue some type of research involvement, albeit for various reasons [12, 17, 20]. Career progression is a primary driver for research activity in medical school [15–17, 20, 21]. In Australia, there is a distinction between students who get involved in research for pragmatic reasons related to gaining a competitive advantage into their preferred clinical specialty, and those with a genuine interest in a research career. Nevertheless, there is much agreement that research during medical school strengthens interest in, and is associated with, future success in an academic career [3, 22].

Also notable are the similarities in perceived barriers to research for a medical student. Time available for research is almost universal [12, 20, 21]. Our data identified ‘Time’ as a major theme that encompassed the connection between age and gender, particularly with females expressing concerns. There is conflicting literature on gender and student research involvement. The systematic review by Amgad et al. [21] found no gender difference in attitudes towards, interest in, or motivation for a research–oriented career, although an international survey [12] found that females were significantly less satisfied with research training during medical school and less likely to pursue it as a career. This is congruent with much of the literature about gender disparity, which is predominantly related to delays in training and longer career interruptions that compete with starting a family [11, 23]. Pathways providing early identification of and encouragement to female students with intentions of a research career, are essential. Additionally, mentoring and guidance from women who have been successful in combining medicine and research should be offered alongside flexible pathways incorporating part-time options for research and clinical training. The Walport model in the UK is a good example of this flexible-functional model of training [24].

Time was also related to the curriculum workload in medical school and the pressure to perform academically that could be compromised by committing to an RHD [12]. A pathway that provides credit for high performing students with flexibility to allow dedicated time for research is worth consideration.

Funding and financial concerns are also widespread among students considering a research-oriented career and our data again connected this to Time [12, 21]. The extra time in research training as either a medical or postgraduate student had negative implications, such as delayed earnings and increased debt.

The theme that encompassed all our findings was Pathway. Collectively, the data herein reflect what is arguably missing in Australia. Australia lacks a national commitment to an organised training pathway to support the outstanding, highly motivated students who genuinely aspire to a clinical research career [25]. This is illustrated in our students’ short- and long-term perceptions of a clinical-research career, which may be likened to a ‘long and leaky pipeline’ of research training [6]. In the short-term, the pipeline suffers from weaknesses in the medical curriculum, which provides little support or flexibility. If students persevere, they are faced with long-term conflicting concerns between managing clinical and research training and the realities of life and family.

Perhaps a pathway strategy for Australia could comprise multiple integrated programs, potentially starting in high school, maintained throughout the medical degree and through the post-graduate years. Medical schools offering an MD-PhD would need to qualify for pathway program support based on merit, making participation highly prestigious and competitive to further drive program excellence. Mentoring and guidance from early in the Bachelor’s degree would help students make informed decisions around their preparation for a PhD in medical school [26]. This early and sustained guidance is vital to keep students engaged, and provide greater certainty around future quality of life, income, and (especially for females) flexible options around childbearing and a family [10].

A clear pathway from undergraduate pre-medical studies, which identified and nurtured genuine potential, would promote the flow of committed and able students through a well-defined MD-PhD program. The pathway would next transition into post-graduate training that is flexible and supportive, allowing further development of skills as junior doctors, but not removed from research training, in order to eventually re-join and enrich younger entries to the pathway through research and teaching.

Recommendations from recent research funding reviews suggest that the time is right to consider new opportunities for solving this problem based on a better understanding of the impediments in Australia. This research needs replication across a larger and more diverse sample that includes high school students through junior doctors and current clinician-scientists, to stimulate a national dialogue and to help devise strategies for piloting pathways at various education levels. Even early outcomes from pilot studies may inform implementation on a larger scale. Whilst there is no quick solution to the dearth of clinician-scientists, Australia’s need to meet the demands of future healthcare compels a more focussed and strategic approach.

### Limitations

Study limitations include a small single institution sample. There is likely sample bias from responders with an interest in research, diminishing the generalizability of the data. Most responders were first-year students, which could have impacted their understanding of the issues. A study strength is the design, allowing us to gather in-depth perceptions from students and qualify the quantitative responses.

## Conclusion

The data emphasise the need for an integrated approach to research training across all stages of medical education. A model that provides a pathway that is flexible and gender equitable, providing appropriate funding, as well as early and consistent mentoring, is likely to have a positive influence on student research career decisions. A national pathway that includes research training along the medical education continuum i.e. medical schools, specialist colleges and health systems, represents a substantial investment for Australia. This investment, rather than a deterrent, should embody Australia’s commitment to ensure our future healthcare is guided by highly trained and competent clinician-scientists.

## Abbreviations

CV: Curriculum Vitae
MD: Medical Doctor degree
MD-PhD: Dual degree program combining an MD and PhD
MPhil: Master of Philosophy Degree which is equivalent to the Research Master of Science Degree
MSTP: Medical Scientist Training Program
NIH: National Institutes of Health
PhD: Doctor of Philosophy
RHD: Research Higher Degree, comprising the PhD and MPhil degrees
US: United States
